# Effector Pt9226 from *Puccinia triticina* Presents a Virulence Role in Wheat Line TcLr15

**DOI:** 10.3390/microorganisms12081723

**Published:** 2024-08-21

**Authors:** Bingxue Wang, Jiaying Chang, Johannes Mapuranga, Chenguang Zhao, Yanhui Wu, Yue Qi, Shengliang Yuan, Na Zhang, Wenxiang Yang

**Affiliations:** 1 Technological Innovation Center for Biological Control of Crop Diseases and Insect Pests of Hebei Province, Hebei Agricultural University, Baoding 071000, China; wangbingxue24@163.com (B.W.); changjiaying@163.com (J.C.); jmapuranga@hotmail.com (J.M.); zhaochenguang@sangon.com (C.Z.); wu203474540@163.com (Y.W.); qiyue@ibcas.ac.cn (Y.Q.); yshl@hebau.edu.cn (S.Y.); 2 Key Laboratory of Plant Resources, Institute of Botany, Chinese Academy of Sciences, Beijing 100093, China

**Keywords:** *Triticum aestivum*, *Puccinia triticina*, effector, transient expressed, virulence

## Abstract

Effectors are considered to be virulence factors secreted by pathogens, which play an important role during host-pathogen interactions. In this study, the candidate effector Pt9226 was cloned from genomic DNA of *Puccinia triticina* (*Pt*) pathotype THTT, and there were six exons and five introns in the 877 bp sequence, with the corresponding open reading frame of 447 bp in length, encoding a protein of 148 amino acids. There was only one polymorphic locus of I142V among the six *Pt* pathotypes analyzed. Bioinformatics analysis showed that Pt9226 had 96.46% homology with the hypothetical putative protein PTTG_26361 (OAV96349.1) in the *Pt* pathotype BBBD. RT-qPCR analyses showed that the expression of Pt9226 was induced after Pt inoculation, with a peak at 36 hpi, which was 20 times higher than the initial expression at 0 hpi, and another high expression was observed at 96 hpi. No secretory function was detected for the Pt9226-predicted signal peptide. The subcellular localization of Pt9226^Δsp^-GFP was found to be multiple, localized in the tobacco leaves. Pt9226 could inhibit programmed cell death (PCD) induced by BAX/INF1 in tobacco as well as DC3000-induced PCD in wheat. The transient expression of *Pt9226* in 26 wheat near-isogenic lines (NILs) by a bacterial type III secretion system of *Pseudomonas fluorescens* EtHAn suppressed callose accumulation triggered by Ethan in wheat near-isogenic lines TcLr15, TcLr25, and TcLr30, and it also suppressed the ROS accumulation in TcLr15. RT-qPCR analysis showed that the expression of genes coded for pathogenesis-related protein TaPR1, TaPR2, and thaumatin-like protein TaTLP1, were suppressed, while the expression of *PtEF-1α* was induced, with 1.6 times at 72 h post inoculation, and TaSOD was induced only at 24 and 48 h compared with the control, when the *Pt* pathotype THTT was inoculated on a transient expression of *Pt9226* in wheat TcLr15. Combining all above, Pt9226 acts as a virulence effector in the interaction between the *Pt* pathotype THTT and wheat.

## 1. Introduction

*Puccinia triticina* (*Pt*) is one of the main pathogens responsible for leaf rust disease in wheat (*Triticum aestivum* L.) [1], which can cause yield losses ranging from 10% to 40% under favorable conditions [2]. *Pt* was found to be the most abundant pathogen present in infected wheat and caused a pandemic in northern plains of China [3]. In recent years, with changing climate conditions, wheat leaf rust disease has become more prevalent in different regions of China [3]. The use of disease-resistant varieties is the most economic, and environmentally friendly, strategy for resistant breeding. The fact is that wheat rust resistance is always “lost” because of the normally single leaf rust resistance gene (*Lr* gene) present in cultivars, which results in high pressure from the *Pt* population. The resistance genes *Lr1*, *Lr2a*, *Lr10*, *Lr14a*, *Lr18*, and *Lr26*, which are common in Chinese wheat cultivars, have lost their resistance to prevalent *Pt* pathotypes [3].

*Pt* is a typical biotrophic fungal plant pathogen that has a unique lifestyle that facilitates the uptake of nutrients from cells of a living host [4]. The successful completion of the life cycle depends on the main host wheat and alternate hosts (*Thalictrum*, *Isopyrum fumarioides*) [5]. In wheat, urediniospores can infect the host repetitively during the growing period, and during the early infection process, haustorium forms from the top of the germ tube, and it mediates the exchange of macromolecules between the pathogen and the host [6]. Effectors are secreted from haustorium and make it suitable for *Pt* growth, and then interfere with the related gene expression of wheat; this is known as effector-triggered susceptibility (ETS) defense action in wheat that is activated by the effectors [7,8]. Effector proteins, as important members of virulence factors, play an important role in this process [8].

Effector proteins are short in length, normally not more than 300 aa [9], cysteine-rich [10], and with a signal peptide on the N-terminal but no transmembrane or conserved domain [11,12]. Some effectors contain motifs such as RxLR in oomycetes [13], Y/F/WxC in powdery mildew fungal [14], [L/I]xAR and YxSL[R/K] in *Magnaporthe oryzae* [15,16], G[I/F/Y][A/L/S/T]R in flax rust [17], and [SG]-P-C-[KR]-P in *Fusarium* [18]. Some other fungal effectors with the LysM motif could inhibit the defense response triggered by chitin [19], and are expressed specifically in tissue or at a particular infection phase [17,20,21,22].

A “zigzag” model was used to interpret the interaction between pathogen invasion and the defense from the plant host, in which the recognition of pathogen-associated molecular patterns (PAMPs) by patten recognition receptors (PRRs) during infection activates PAMP-triggered immunity (PTI) [8]. Plant pathogens secrete virulence factors called effectors into the host cells to suppress PTI components [23]. The effectors, which are specific to certain pathogen races or species, target a variety of host plant proteins and metabolic pathways to manipulate the physiological state and defense response of the host, as well as host components to enhance pathogen virulence [24,25]. In response, host plants evolved another layer of immunity, in which a large array of receptors often organized to recognize the effector directly or indirectly and activate a strong immune response called effector-triggered immunity (ETI) [26,27]. This results in a series of induced mechanisms, including the bursting of reactive oxygen species (ROS) and the hypersensitive response (HR) accumulation of pathogenesis-related proteins (PRs) and phytoalexins [28]. In this process, it is important for the pathogen to avoid host recognition to overcome the defense response of the host.

*Puccina triticina* is one of the obligatory parasites that damage wheat production. Exploring the *Pt* virulence effector proteins and their functions during *Pt* infection can provide a theoretical basis for the development of wheat cultivars with durable disease resistance, which will help to control wheat leaf rust. In our previous study, a candidate effector, Pt9226, which was differentially expressed between three pathotypes of KHTT, JHKT, and THSN, was screened using bioinformatic analysis. To better understand the molecular mechanisms underlying the role of Pt9226 during infection, in this study, we reported the gene cloning of Pt9226 and its expression profile during *Pt* infection. The virulence function was analyzed using the transient expression of *Pt9226* in *Nicotiana benthamiana* and in wheat. This study unravels the molecular mechanisms underlying the inhibition of the basic defense response of the wheat host by *Pt9226*, and provides a foundation for further revealing the regulation mechanism of *Pt* effectors.

## 2. Materials and Methods

### 2.1. Plant Materials, Pt Pathotypes, and Primers

Twenty-six near-isogenic lines (NILs) in a Thatcher background (TcLr1, TcLr2a, TcLr2b, TcLr2c, TcLr3ka, TcLr3bg, TcLr9, TcLr10, TcLr14a, TcLr14b, TcLr15, TcLr17, TcLr20, TcLr21, TcLr23, TcLr25, TcLr26, TcLr28, TcLr29, TcLr30, TcLr32, TcLr33, TcLr36, TcLr41, TcLr42, and TcLr44) were kindly provided by Centro International de Mejoramientode Maizy Trigo (CIMMYT). The plant materials were maintained in a wheat leaf rust disease center at Hebei Agricultural University. Vermiculite: humus soil (1:1) was prepared as a culture substrate. Wheat lines were planted in plastic pots (9 cm) and grown in a greenhouse at 10–22 °C, 16 h light/8 h darkness. The 26 NILs were utilized for the screening of the potential interaction target of effector Pt9226, out of which TcLr15 was used for further analysis based on the results.

*Pt* pathotypes 08-5-361-1 (THTT), 09-19-284-1 (THTS), 03-5-99 (PHTP), 08-5-11-1 (FHHT), 08-5-260-2 (THKT), and 08-5-9-2 (KHHT) were collected from a field population and purified and identified according to Long and Kolmer [29]. All experiments were performed simultaneously with three replicates.

All the primers used in this study were synthesized by Sangon Biotech (Shanghai) Co., Ltd. (Shanghai, China) and are listed in Supplementary Table S1.

### 2.2. Cloning and Bioinformatic Analysis of Pt9226

A full diseased wheat plant inoculated with each *Pt* pathotype was used to extract the genomic DNA by using the CTAB method [30]. DNA samples were stored at −20 °C before use. The coding sequence of *Pt9226* was amplified from the gDNA of the 6 *Pt* pathotypes using the primer pair Pt9226-F and Pt9226-R, following the protocol of Zhang [31] except for the annealing temperature of 60 °C. The purified PCR amplicons were ligated into a pMD19-T vector (TaKaRa), transformed into *Escherichia coli* DH5α by heat shock transformation, and correct clones, which were sequenced by Sangon Biotech (Shanghai) Co., Ltd. The acquired sequences were aligned with MEGA 7 for polymorphism analysis. Sequence analysis was conducted using BLAST (http://www.ncbi.nlm.nih.gov/blast/, accessed on 6 January 2022.), CD-search (http://www.ncbi.nlm.nih.gov/Structure/cdd/wrpsb.cgi, accessed on 6 January 2022), and ORF finder (http://www.ncbi.nlm.nih.gov/orffinder/, accessed on 6 January 2022). Motif analysis was performed using MEME FIMO 5.5.2. The signal peptide was predicted by SignalP 4.1 (http://www.cbs.dtu.dk/services/SignalP/, accessed on 6 January 2022). TargetP 2.0 Serve combined with Cell-PLoc 2.0 (http://www.csbio.sjtu.edu.cn/bioinf/Cell-PLoc-2/, accessed on 6 January 2022) was used for subcellular localization prediction. Protscale (http://web.expasy.org/protscale, accessed on 6 January 2022) was used for the analysis of the hydrophilic/hydrophobic scales. SOPMA (https://npsa-prabi.ibcp.fr/cgi-bin/npsa_automat.pl?page=/NPSA/npsa_sopma_f.html, accessed on 6 January 2022) and SWISS-MODEL (http://swissmodel.expasy.org/, accessed on 6 January 2022) were used for protein structure prediction.

### 2.3. Plant Materials Treatments

For the expression profile of Pt9226 by RT-qPCR, TcLr15 wheat seedlings were inoculated with THTT when the first leaves were fully expanded. Samples were collected at 0, 6, 12, 18, 24, 36, 48, 72, 96, 144, and 216 h post inoculation (hpi). For the expression of disease resistance-related genes, wheat leaves transiently expressing pEDV6-Pt9226 and pEDV6 were inoculated with the Pt pathotype THTT. The samples were collected at 0, 12, 24, 48, and 72 h after Pt inoculation. The samples were quickly frozen in liquid nitrogen and stored at −80 °C before use, with three replicates for each time point.

Total RNA was extracted according to BIOZOL Total RNA Extraction Reagent (BioFlux, Beijing, China) instruction. cDNA was synthesized using Reverse Transcriptase M-MLV (RNase H^−^) (abm) according to the manufacturer’s instructions. The synthesized cDNA samples were stored at −20 °C for further use.

### 2.4. RT-qPCR Analysis of the Gene Expression

The RT-qPCR was performed by using 2 × TransStart^®^ Top Green qPCR SuperMix (+Dye I) (TransGen Biotech., Beijing, China) on (CFX connect, BIO-RAD). The reaction was performed as follows: 94 °C for 30 s, followed by 30 cycles of 5 s at 94 °C, 15 s at 60 °C, and 10 s at 72 °C.

The complementary DNA (cDNA) was used as a template to analyze *Pt* biomass using EF1-α as the target gene, with *Pt actin* gene as the reference gene. Meanwhile, the same cDNA was taken as a template to analyze the expression of diseases’ resistance-related genes (*TaPR1*, *TaPR2,* and *TaTLP1*) and superoxide dismutase (SOD) in wheat, with *TaGAPDH* as a reference gene (Supplementary Table S1). The relative expression level of the target gene was presented as the fold change compared with the internal control using the 2^−ΔΔct^ method, and data were analyzed with DUNCAN’s multiple range test (*p* < 0.05). Three biological replicates were performed for each test.

### 2.5. Yeast Secretion Assay

The functional validation of the predicted signal peptide was performed using the yeast secretion assay following the protocol by the authors of [32]. In short, Pt9226^sp^ was amplified from the recombined vector pMD19-T:Pt9226 using primer Pt9226FM-F and Pt9226FM-R. Purified Pt9226^sp^ amplicon and pSUC2T7M13ORI (pSUC2 with a truncated invertase gene *SUC2*, lacking its own signal peptide sequence and the fist codon) were digested by *EcoR* I and *Xho* I and ligated to construct pSUC2-Pt9226^sp^. Then, pSUC2-Pt9226^sp^ was transformed into the invertase-deficient yeast strain YTK12. The secretory function was determined by the growth of YTK12 on YPRAA media (amended with Antimycin A). pSUC2-Mg87 and pSUC2-Avr1b were used as negative and positive controls, respectively. pSUC2-Mg87, pSUC2-Avr1b, and pSUC2-Pt9226^sp^ in YTK12 were transferred into SD/-Trp, and YTK12 was transferred into YPDA and cultured at 28 °C at 200 rpm for 24 h, spun for 5 min at 12,000 rpm to collect the cells, and OD_600_ was adjusted to 0.6 using sterile water. TTC-NaOH was then used (*m*/*v*: 0.05%) as the color-substrate solution. The invertase enzyme activity was detected by its ability to reduce 2,3,5-triphenyltetrazonium chloride (TTC) to insoluble red 1,3,5-triphenylformic acid (TPF).

### 2.6. Subcellular Localization of Pt9226^△sp^ in Nicotiana Benthamiana Leaves

pBI121-Pt9226^△sp^-GFP was constructed and transformed into *Agrobacterium tumefaciens* GV3101 competent cells. After culturing in LB (amended with 50 μg·mL^−1^ kanamycin and 25 µg·mL^−1^ rifampicin) at 28 °C at 200 rpm for about 2 d, the solution was spined for 5 min at 1000 rpm, rinsed twice with sterile water, and the OD_600_ was adjusted to 0.5 with buffer. The recombinant fusion protein was then infiltrated into *N. benthamiana*. The GFP signal was detected using a confocal microscope OLYMPUS BX51 (Olympus Corporation, Tokyo, Japan).

### 2.7. Transient Expression of Pt9226 in N. benthamiana

pGR107-Pt9226^△sp^-GFP was constructed and transformed into GV3101 competent cells. It was then cultured in Luria-Bertani medium containing 25 mg·L^−1^ rifampicin and 50 mg·L^−1^ kanamycin. GV3101 carrying the corresponding construct was suspended in *Agrobacterium* buffer, and strains with an OD_600_ of 0.5 were then infiltrated into *N. benthamiana* leaves. pGR107-BAX and pGR107-INF1 were infiltrated 24 h later on the same spot. The vector pGR107-GFP was used as a blank control.

### 2.8. Transient Expression of Pt9226 in Wheat by Bacterial Type III Secretion System

pEDV6-Pt9226 was constructed and transformed into *EtHAn* competent cells. Then, *EtHAn* was infiltrated into the second wheat leaves, with the empty pEDV6 in *EtHAn* used as a negative control. Samples were collected at 24, 48, and 72 h post infiltration. Callose deposition was observed using a fluorescence microscope (Nikon, Tokyo, Japan). In short, the samples were thoroughly discolored by 75% ethyl alcohol, then bleached with 0.5 mol·L^−1^ NaOH for 10 min and rinsed with sterile water, and then with 0.067 mol·L^−1^ K_2_HPO_4_ for 30 min. The prepared leaf segments were stained in 0.05% aniline blue, rinsed, and then stored in 20% glycerol before microscopy. Reactive oxygen species (ROS) were observed using the fluorescence microscope (Nikon, Japan). The leaf segments were immersed in 10 mg/mL Diaminobenzidine (DAB) and exposed to heavy light for 8~10 h, then were discolored in absolute ethyl alcohol/glacial acetic acid (1:1), and rinsed with sterile water to wipe off the retained destaining solution by the authors of [33]. Each time point had three replicates and five microscope fields for each leaf we observed. Image-Pro Plus 6.0 was employed to calculate the area of reaction.

### 2.9. Data Analysis

All experimentation was performed with three repetitions. SPSS 21.0 software was used for statistical analyses. Duncans’ new multiple-range test, at the *p* < 0.05 or *p* < 0.01 level of significance, was used to compare the differences among the treatments.

## 3. Results

### 3.1. Pt9226 Is Highly Conserved in Pt Pathotypes

The amplified *Pt9226* gene sequence was 887 bp (accession No. PQ001671) at the genome level, containing six exons and five introns, and the full length of the ORF sequence at the RNA level was 447 bp. A polymorphism analysis of the ORF sequences of Pt9226 in six *Pt* pathotypes, THTT, THTS, PHTP, FHHT, THKT, and KHHT, revealed that there was only one polymorphic site. The *Pt9226* sequence encodes a protein sequence of 148 amino acids. In the amino acid sequences of physiological subspecies PHTP, FHHT, THKT, and KHHT, the 142nd amino acid residue of the Pt9226 protein sequence is valine (V), while in THTT and THTS, this site is isoleucine (I), suggesting that Pt9226 is highly conserved in *Pt* pathotypes. Online prediction showed that the two protein sequences had the same tertiary structure, suggesting that this single amino acid change does not affect the higher-level structure or the possible function of the protein.

The Pt9226 protein sequence had no conserved domain or fungal motif. The predicted molecular weight was 16.13 KDa, and the theoretical isoelectric point was 6.37. It belongs to a hydrophilic protein (HI: −0.215) and a labile protein with an index of 40.85. The signal peptide (SP) cleavage site was between 21 and 22 amino acids, implying a 21aa SP. No transmembrane domain was predicted. Based on the Cell-PLoc 2.0 results and further analysis with TargetP 2.0, we deduced that Pt9226 was a nucleus-localized protein. A homologous sequence search by NCBI revealed that the Pt9226 protein sequence had a homology of 96.46% with the hypothetical protein PTTG_26361 (OAV96349.1) from the *Pt* pathotype BBBD, and a homology of 40.00% to 54.24% with some hypothetical proteins from *P. striiformis* f. sp. *tritici* (*Pst*) and *P. graminis* f. sp. *tritici* (*Pgt*). A phylogenetic tree was generated using the homologous amino acid sequences of Pt9226, downloaded from the NCBI database (Figure S1).

### 3.2. Pt9226 Is Induced during Pt Infection in Wheat

**Figure 1:**
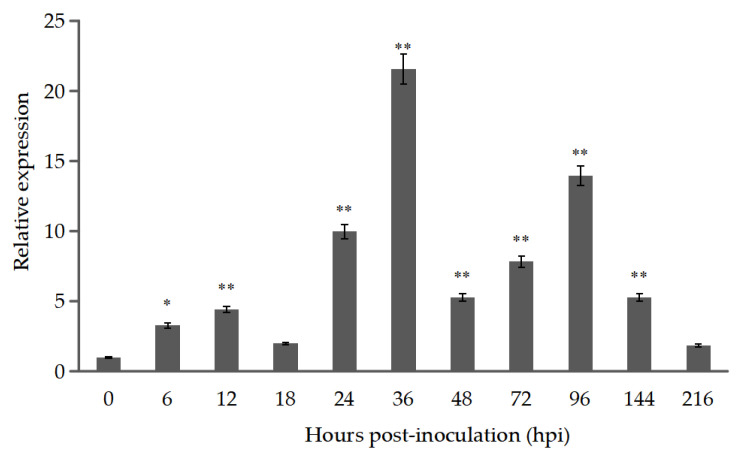
The expression pattern of *Pt9226* in the wheat line TcLr15 after inoculation with the *Pt* pathotype THTT. The relative gene quantification was calculated by the comparative *Ct* method, with *Pt*-actin as the reference gene, and was relative to that of 0 hpi. The expression at 0 h post-inoculation was standardized as 1 (*: *p* < 0.05, **: *p* < 0.01).

RT-qPCR analysis showed that *Pt9226* expression was significantly increased at 24 h after *Pt* inoculation, reaching 11 times the initial expression level, and it reached a peak at 36 h, which was 23.7 times higher than the initial expression level. By 48 hpi, the expression level decreased significantly, and later increased at 96 h (Figure 1). The results suggest that Pt9226 may play an important role during *Pt*-wheat interaction, especially during the early stages of infection.

### 3.3. Signal Peptide of Pt9226 Has No Secretory Function

**Figure 2:**
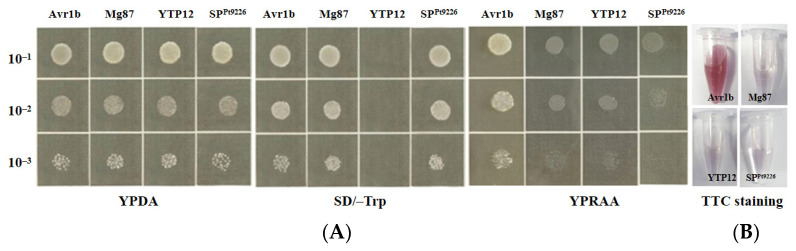
The signal peptide of Pt9226 has no secretary function. (**A**) Signal peptide secretion function identification of SP^Pt9226^ using the yeast invertase secretion assay. (**B**) Signal peptide secretion function identification of SP^Pt9226^ stained with TCC. Avr1b (positive control), Mg87 (negative control), YTK12 (blank control), and SP^Pt9226^ (signal peptide of Pt9226), respectively.

The predicted SP sequence of Pt9226 (SP^Pt9226^) was fused with pSUC2 to construct pSUC2-SP^Pt9226^ to help the *SUC2* gene acquire the complementary SP so it can secrete the encoded invertase. This was followed by the transformation of the fusion construct into YTK12, an invertase deficient strain. YTK12 (blank control), pSUC2-Avr1b (positive control), pSUC2-Mg87 (negative control), and pSUC2-SP^Pt9226^ grew well on SD/-Trp, indicating that the corresponding Avr1b, Mg87, and SP^Pt9226^ have been successfully transformed. On the YPRAA medium, pSUC2-SP^Pt9226^ could not grow, which was consistent with pSUC2-Mg87 (Figure 2A), indicating that SP^Pt9226^ has no secretory function. This was further verified by 2,3,5-Triphenyl-2H-tetrazolium chloride (TTC) to detect the enzyme activity, but no brick-red color was observed in the reaction tube. These results suggest that the Pt9226 putative N-terminal signal peptide has no secretory function (Figure 2B).

### 3.4. Pt9226 Is a Multiple Subcellular Compartments-Localized Protein

**Figure 3:**
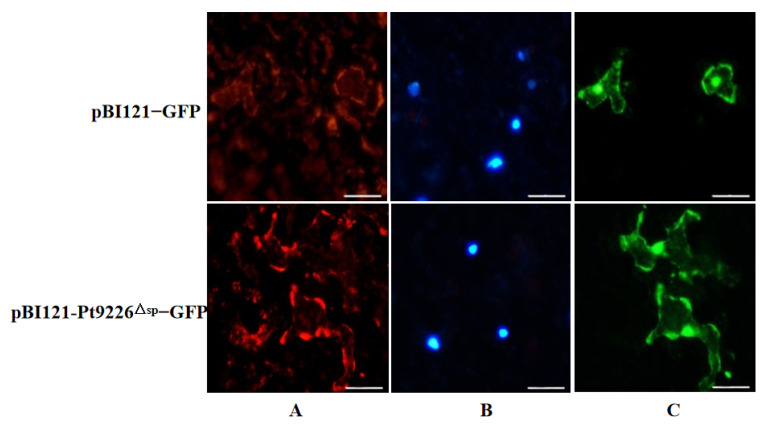
Pt9226 is a multiple subcellular localized protein in *N. benthamiana*. (**A**) The cell membrane is shown in red; (**B**) the nucleus is shown in blue; (**C**) the GFP is shown in green fluorescent. Scale bar = 100 μm

The *Agrobacterium*-mediated transient expression was proceeded in *N. benthamiana* leaves with pBI121-Pt9226^△sp^-GFP, which was cultured in darkness at 25 °C for 24 h. Fluorescence was observed under 488 nm 48 h after infiltration. Several subcellular compartments of the infiltrated *N. benthamiana* cells exhibited green fluorescence (Figure 3): the recombinant Pt9226-GFP without the SP showed a similar localization in the transient expressed *N. benthamiana* leaves (Figure 3), indicating that Pt9226 accumulates in the periplasmic regions, probably in many subcellular compartments of the plant cells.

### 3.5. Pt9226 Suppressed Cell Death Induced by BAX and INF1 in N. benthamiana

**Figure 4:**
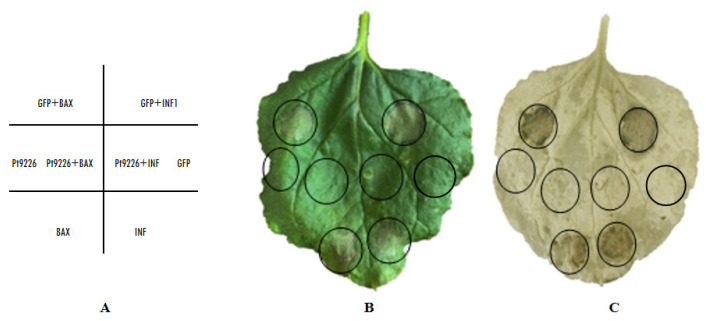
Pt9226 suppressed BAX/INF1−induced cell death in *N. benthamiana.* (**A**) The schematic diagram of infiltration. (**B**) The *Agrobacterium* GV3101 carrying PVX:Pt9226 and PVX:GFP were infiltrated in *N. benthamiana*, and after 24 h, *Agrobacterium* carrying PVX:BAX or PVX:INF1 were infiltrated on the corresponding positions. No cell death was induced by BAX and INF1 24 h after infiltration with *Agrobacterium* carrying PVX:Pt9226. (**C**) The decolorized phenotype.

Pro-apoptotic mouse protein BAX and the *Phytophthora infestans* elicitin INF1 triggered cell death symptoms in plants which are same as those related to the immunity-associated hypersensitive response [34]. BAX and INF1 were infiltrated, respectively, into the *N. benthamiana* leaves 24 h after pre-infiltration with pGR107-Pt9226^△sp^-GFP and pGR107-GFP (blank control). Necrosis formed on leaves 24 h after infiltrated with BAX and INF1 indicated that they triggered cell death on tobacco leaves, while no necrosis formed on pGR107-Pt9226^△sp^-GFP pre-infiltrated leaves, indicating that Pt9226 showed no virulence to tobacco, and the transient expression of Pt9226 suppressed BAX-/INF1-triggered cell death (Figure 4). GFP expression in *N. benthamiana*, as a negative control, did not suppress cell death triggered by BAX/INF1. All these results indicate that Pt9226 suppresses the immunity-associated response in *N. benthamiana.*

### 3.6. Pt9226 Suppresses Cell Death in Wheat Leaves

**Figure 5:**
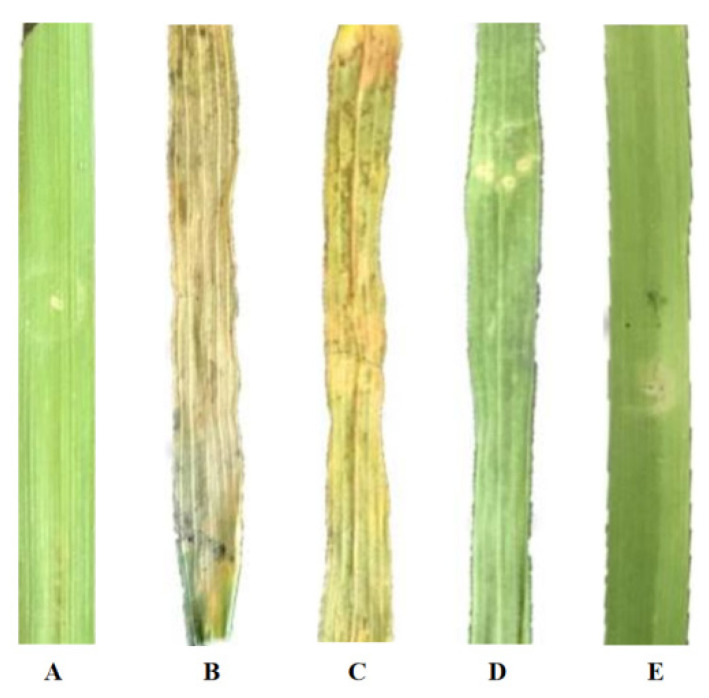
Pt9226 suppressed cell death triggered by DC3000 in wheat. The phenotypes of wheat leaves infiltrated with (**A**) pEDV6; (**B**) DC3000; (**C**) DC3000 + pEDV6; (**D**) Pt9226; and (**E**) DC3000 + Pt9226.

The constructed pEDV6-Pt9226 and empty vector of pEDV6 were transformed into an EtHAn competent cell, and the cultured strains accompanied with *Pseudomonas syringae* pv. *tomato* (DC3000) were infiltrated into wheat leaves in a ratio of 1:1. No visible necrosis appeared on leaves infiltrated with pEDV6-Pt9226 (Figure 5A), and DC3000 induced a hypersensitive response (HR) in wheat leaves (Figure 5B). Necrosis formed on leaves co-infiltrated with pEDV6 and DC3000 indicated that an HR induced by DC3000 could not be suppressed by the pEDV6 vector (Figure 5C). No visible HR formed on leaves infiltrated with pEDV6-Pt9226 (Figure 5D) or pEDV6-Pt9226 accompanied with DC3000 (Figure 5E), indicating that Pt9226 can suppress cell death triggered by DC3000 in wheat.

### 3.7. Transient Expression of Pt9226 Reduced Callose Deposition and ROS Accumulation in Wheat Leaves

**Figure 6:**
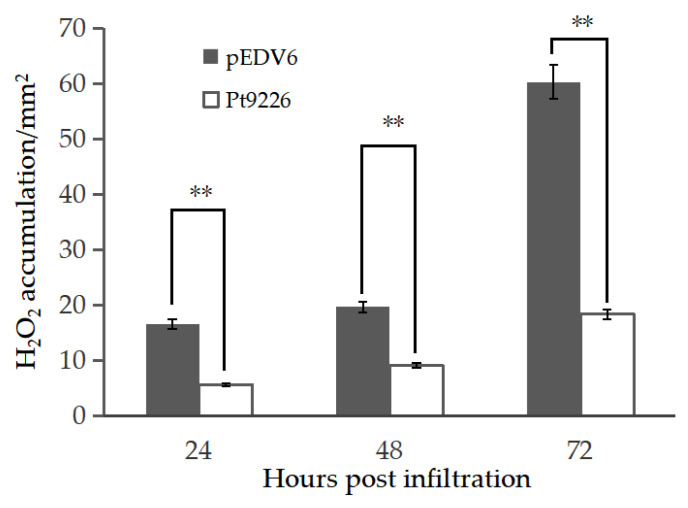
Overexpression of *Pt9226* in wheat cells suppressed reactive oxygen species (ROS) accumulation. Inoculated leaves were sampled at 24, 48, and 72 hpi and stained with 3,3′-diaminobenzidine for detection of ROS. Staining area was calculated using Image-Pro Plus 6.0 software, and area of ROS was measured in pixels. ROS accumulation was suppressed in TcLr15 expressing pEDV6-Pt9226 compared with blank control (**: *p* < 0.01).

The virulence of pEDV6-Pt9226 was further analyzed using the bacterial type III secretion system, and we observed reduced callose presented in wheat near isogenic lines TcLr15, TcLr25, and TcLr30, but there was no difference in most wheat materials compared to pEDV6 (blank control). These results indicate that Pt9226 can suppress callose deposition induced by EtHAn in TcLr15, TcLr25, and TcLr30, with suppression ratios of 55.7%, 25.4%, and 36.8%, respectively (Supplementary Figure S2). Reactive oxygen species (ROS) accumulation was increased both in TcLr15 plants infiltrated with pEDV6-Pt9226 and the blank control pEDV6, but it was suppressed by 66.3%, 53.8%, and 69.5%, respectively, at 24 h, 48 h, and 72 h after inoculation in TcLr15 infiltrated with pEDV6-Pt9226 (Figure 6).

### 3.8. Transient Expression of Pt9226 in TcLr15 Reduced Resistance to Pt

**Figure 7:**
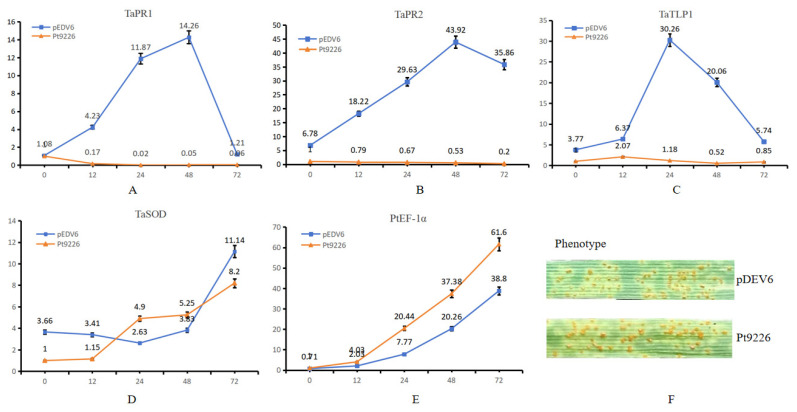
Expression profiles of pathogenesis-related genes and the phenotypic observation of TcLr15 14 days post inoculation with THTT. (**A**–**E**) Expression profiles of *TaPR1*, *TaPR2*, *TaTLP*, *TaSOD*, and *PtEF1-α*, respectively. The expression of *TaPR1*, *TaPR2*, and *TaTLP1* was suppressed significantly, while *TaSOD* was induced. The expression of these genes was detected in pDEV6-Pt9226-infiltrated TcLr15 plants, with the vector pDEV6 as a blank control. (**F**) the phenotype of the plant infiltrated with pEDV6 and pEDV6-Pt9226 14 d after THTT inoculation. The biomass of *Puccinia triticina* increased in TcLr15 plants after being inoculated with the *Pt* pathotype THTT, and the phenotype changed to “4” in Pt9226 transiently expressed TcLr15 compared with the control plants (“3”).

The *Pt* pathotype THTT (08-5-361-1) was inoculated on the pDEV6-Pt9226 transiently expressing wheat line TcLr15 24 h after infiltration. RT-qPCR (Quantitative Real-time PCR) was used to analyze the expression of pathogenesis-related genes *TaPR1*, *TaPR2*, and *TaTLP1*, and the ROS-related gene (*TaSOD*), as well as the expression level of wheat leaf rust pathogen elongation factor *EF1-α*, and the expression level of each gene at 0 h in the experimental group was standardized to 1. The expression profiles of *TaPR1*, *TaPR2*, and *TaTLP1* showed an initial increase followed by a decrease, reaching their expression peak at 48 h, and 24 h, respectively, in the samples collected from pDEV6 (blank control), while the expression of the three genes seemed to be extremely suppressed in pDEV6-*Pt9226* pre-infiltrated TcLr15 leaves (Figure 7). The expression of *TaSOD* was upregulated in Pt9226 transiently expressing plants (Figure 7). *PtEF1-α* expression was induced in both pDEV6-*Pt9226* transiently expressing plants and the control from 0 h to 72 h, with a relatively high expression in pDEV6-Pt9226 pre-infiltrated leaves (Figure 7).

The phenotypic observation of TcLr15 14 days after inoculation showed a massive uredospore development on pEDV6 (IT3) and a relative larger uredinium on pEDV6-Pt9226 (IT4) pre-infiltrated leaves (Figure 7).

## 4. Discussion

The understanding of the molecular mechanisms underlying host-pathogen interactions is critical for the development of effective disease control strategies [35,36]. The emergence of new pathogen races with high virulence causes significant challenges [37]. *Pt* is highly variable because the virulent races can rapidly undergo evolutionary changes to circumvent resistance in wheat cultivars. Genes encoding effectors are among the most rapidly evolving genes in pathogen genomes [38], and this results in the considerable diversity of effectors at the genus and species level [38,39,40]. But in the present study, Pt9226 showed low polymorphism within different *Pt* virulent pathotypes, indicating that Pt9226 is a conserved protein that may play an important role during *Pt*-wheat interaction.

Effector proteins often have some common characteristics, such as the presence of a signal peptide with a secretory function at the N-terminus, and which are rich in cysteine [41]. Studies have shown that the signal peptide and abundant cysteine are related to the secretion and transport of effector proteins to the specific sites for pathogen-host interaction [42,43]. However, there are also signal peptides of effector proteins that do not have a secretory function [41], implying that these effector proteins may be secreted through other pathways (such as the bacterial type III secretion system, etc.) [44]. Some effector proteins contain some special protein motifs that play an important role in the invasion of pathogens, such as RxLR, RxLR-like, and LFLAK-HVLVxxP (CRN) motifs, which can help effector proteins enter host cells [12]. In addition, effector proteins with Y/F/WxC motifs at the N-terminus have been found in obligate pathogens, such as barley powdery mildew, wheat stripe rust, and wheat leaf rust [22], but their specific role in the function of haustoria is not yet clear. The N terminal Y/F/WxC motif downstream of the SP was conserved in 80% of the candidate-secreted effectors in powdery mildew fungi [14]. The flax rust *Melampsora lini* effector protein AvrM was shown to be translocated into the host-cell in a pathogen-independent manner, where it bound to phosphoinositides and was directly recognized by *Linum usitatissimum* disease resistance M, resulting in the activation of effector-triggered immunity [45]. The Bacterial type III secretion system (T3SS) proved to be effective in delivering pathogen proteins into the host plant cells [46,47].

There are also some other non-classically secreted effectors lacking the N-terminal signal peptide that are secreted by a distinct secretion pathway [48,49]. These effectors, such as *Phytophthora sojae* effector PsIsc1 and *Verticillium* dahliae effector VdIsc1, are secreted as isochorismatases and are essential for full pathogenesis [50]. Another non-classically secreted *Magnaporthe oryzae* effector MoNte1 that is translocated into the host nuclei mediated by a nuclear targeting peptide is crucial for invasive fungal growth, appressorium formation, and host colonization [49]. In this study, Pt9226 showed no conserved motif, the signal peptide had no secretory function, and it was found to be a multiple subcellular compartment-localized protein, suggesting that it has a specific molecular mechanism in the *Pt* pathogenic process. The lack of a functional signal peptide suggests that it might be a non-classical effector protein that is secreted by a unique pathway that needs to be further explored in the future.

Most effectors are expressed periodically in appressorium or haustorium, or specifically expressed in the infected tissues of the host. During pathogen infection, the expression profile can be used to predict the role of the effector. *Pt9226* showed a high level of transcription during *Pt* infection, especially from 24 to 36 hpi, which is the critical period for the formation of substomatal vesicles and haustorium, and with another expression peak at 96 hpi, during which massive hyphae and uredospores are formed, indicating that Pt9226 is a key virulence factor during *Pt* infection.

During *Pt*-wheat interaction, callose deposition was considered as one of the characteristics of defense system operation in PTI [51], while the hypersensitive reaction (HR) was presented as the main response during ETI, in which intracellular immune receptors (NLRs) were recognized by the effector [52]. The physiological characteristics of programmed cell death (PCD) induced in plants are similar to those of the HR triggered by pathogens [53,54]. ROS play an important role in both PTI and ETI, and ROS bursting depends on the sustained activation of the PRRs pathway [55,56]. For example, PstGSRE, a glycine-serine-rich effector from *Pst*, interacts with wheat LOL2 (lesion simulating disease 1 (LSD1)-like zinc finger protein) to interfere with the nuclear transport of LOL2 and LOL2-regulated ROS-stimulated defense gene expression [9]. Some effectors can also activate host susceptibility pathways, which benefit the pathogen [57,58,59]. The *Pt* effector Pt13024 was found to be avirulent to TcLr30, and it induced callose deposition and ROS bursting [60]. As an avirulence effector, Pt18906 can stimulate the accumulation of callose and the bursting of ROS in wheat line TcLr27+31, thus it triggered the two-layer defense reaction of TcLr27+31 [33]. PTTG_08198 also promoted ROS accumulation, indicating that it is an avirulence effector [61]. Another *Pt* avirulence protein, AvrLr15, that triggers *Lr15*-dependent immunity was recently identified, and it was found that mutations in four amino acids in the AvrLr15 proteins result in a loss of virulence [62]. However, in this study, both callose deposition and ROS bursting triggered by DC3000 were suppressed by the transient expression of *Pt9226* in wheat near isogenic line TcLr15, suggesting that Pt9226 enhances *Pt* virulence by suppressing wheat PTI responses. Pt9226 is the first candidate effector that presents virulence to a specific *Lr* gene. The pathogenic mechanism and the target of Pt9226, as well as how it influences the resistance of TcLr15, is to be further explored. Overall, this study provides novel insights into the molecular mechanisms underlying *Pt* infection, and it lays a foundation for the development of new strategies to control wheat leaf rust.

## 5. Conclusions

The candidate effector Pt9226 was cloned from the genomic DNA of the *Pt* pathotype THTT. This gene could inhibit PCD induced by BAX/INF1 in tobacco as well as DC3000-induced PCD in wheat. Pt9226 acts as a virulence factor in the interaction between the *Pt* pathotype THTT and wheat. These findings provide molecular insights into the *Pt*-wheat interaction, and theoretical bases of the protection of wheat against *P. triticina*.

## Data Availability

This manuscript includes the necessary data, either as figures, tables or as Supplementary Materials. The data is available on request from the corresponding authors.

## Supplementary Materials

The following supporting information can be downloaded at https://www.mdpi.com/article/10.3390/microorganisms12081723/s1, Figure S1: Phylogenetic tree of effector Pt9226 from *Puccinia triticina*, Figure S2: Callose area statistics of Pt9226 in 26 near-isogenic lines. Table S1: Primers and their sequences used in the research.

## Abbreviations

Pt, *Puccinia triticina*; *Lr*, leaf rust-resistance gene; ETS, effector-triggered susceptibility; NIL, near-isogenic line; PRRs, pattern-recognition receptors; PAMPs, pathogen-associated molecular patterns; PTI, PAMP-triggered immunity; PCD, programmed cell death; ROS, reactive oxygen species; EF, elongation factor; and SOD, super oxide dismutase.
