# Supplementary material for: Effector Pt9226 from Puccinia triticina Presents a Virulence Role in Wheat Line TcLr15
Source: Microorganisms. 2024 Aug 21;12(8):1723. doi: 10.3390/microorganisms12081723 (PMC11357290; doi:10.3390/microorganisms12081723)
Supplement: Supplementary file 1 [file microorganisms-12-01723-s001.zip › microorganisms-3150920-supplementary.pdf]

Supplementary Materials

Table S1. Primers and their sequences used in the research.

Primer Name	Primer Sequence (5′–3′)	Related Experiment
Pt9226-F	ATGACAGCCCGCCCGCTTT	Gene cloning
Pt9226-R	TCAGGCTAACGTGAACCCCC	
qPt9226-F	GCTTCAGCCACTACCGCA	
qPt9226-R	AACCCCCCTATTATCTCATCG	
qPtACT-F	CCGCCTTGGTTCTTGACAATGGTT	
qPtACT-R	ATTCCGACCATCACACCCTGATGA	
qTaGAPDH-F	CTGCCTTGCTCGTCTTGCTAA	
qTaGAPDH-R	CTTGATGGAAGGACCATCAAC	
qTaSOD-F	CCGAGGTCTGGAACCATCAC	Quantitative real-time PCR
qTaSOD-R	AGCCGAAATCCTTCTCGATCT	
qTaPR1-F	CAATAACCTCGGCGTCTTCATC	
qTaPR1-R	ATTTACTCGCTCGGTCCCTC	
qTaPR2-F	CAACGAGAACCAGAAGGACAGC	
qTaPR2-R	TACGGACGGACATACGGACACT	
qTaTLP1-F	GGGATCCATGGCGACCTCCGCGGTGCTC	
qTaTLP1-R	CCAAGCTTTCATGGACAGAAGGTGATCTGGTC	
Pt9226PZ-F	*caccATGTTAGGCTTCACGCAGCT	
Pt9226PZ-R	GGCTAACGTGAACCCCCCTA	Transient expression in wheat
M13-pEntry-F	GTAAAACGACGGCCAG	
M13-pEntry-R	CAGGAAACAGCTATGAC	
Pt9226FM-F	**ccggaattcATGTTAGGCTTCACGCAGC	Signal peptide secretion function
Pt9226FM-R	**ccgctcgagGATCATTCCATGATGTAGGAGG	
Pt9226pBI-F	***catttacgaacgatactcgagATGACAGCCCGCCCGCTTT	Subcellular localization
Pt9226pBI-R	***caccatcactagtacgtcgacGGCTAACGTGAACCCCCCTA	

Note: *, "cacc" is Kozak sequence which is required for gene expression in eukaryotic; **, protective bases "ccg" and enzyme cutting sites of *EcoR* I (gaattc) and *Xho* I (ctcgag); ***, homologous sequence with the vector.

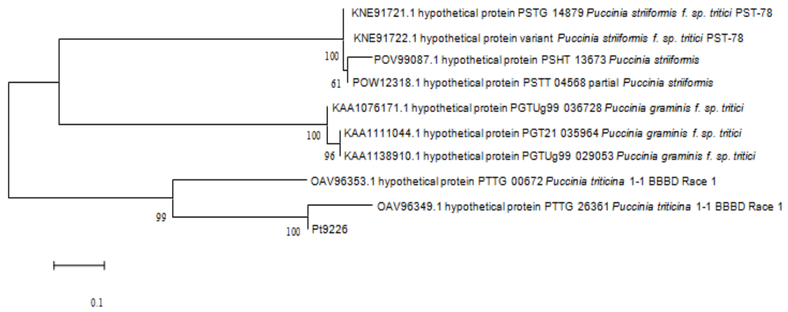

Figure S1. Phylogenetic tree of effector protein Pt9226 from *Puccinia triticina*. Pt9226 showed high identity with hypothetical protein PTTG 26361 (OAV96349.1) from *Puccinia triticina* race 1 (BBBD)

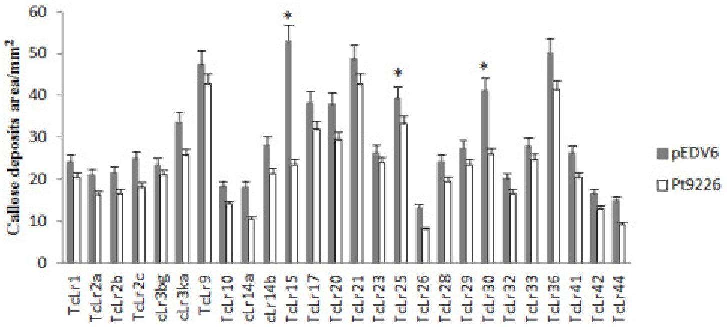

Figure S2. Callose deposition after overexpression of Pt9226 in 26 near-isogenic lines. * Callose deposition were suppressed with significant difference in line TcLr15, TcLr25, and TcLr30.
